# Singleness, Religiosity, and the Implications for Counselors: The Indonesian Case

**DOI:** 10.5964/ejop.v14i2.1530

**Published:** 2018-06-19

**Authors:** Karel Karsten Himawan, Matthew Bambling, Sisira Edirippulige

**Affiliations:** a Faculty of Medicine, University of Queensland, Brisbane, Australia; b Faculty of Psychology, Universitas Pelita Harapan, Tangerang, Indonesia; c Centre for Online Health, University of Queensland, Brisbane, Australia; Department of Psychology, Webster University Geneva, Geneva, Switzerland

**Keywords:** singles, never-married, religiosity, well-being, Indonesia, counseling

## Abstract

This paper explores the unique role of religiosity in assisting Indonesian singles (extensively refer to those who are never married) and how it relates to the counseling and therapeutic practices with never-married clients. Whereas the role of religiosity has been drawn into scholarly attention for its effectiveness in dealing with many situations that are particularly related to social stigma, little is known regarding its role in assisting singles to overcoming stigma due to their singleness. Indonesian society regards marriage as a social achievement and this perception places singles in an undervalued position. On the contrary, the society regards positively those who demonstrate religious attributes. Therefore, religiosity is a potential factor that mediates social perception of singles who attach themselves to religious attributes (such as: religious symbols and rituals). Using database search methodology, this paper presents an overview of how religiosity assists singles in overcoming their challenges and discusses the implications of those dynamics in counseling settings.

Human beings desire happiness, and therefore they direct their every action toward achieving it (Emmons, 2005). Many personality theorists explain how individuals develop certain mechanisms to achieve and maintain their happiness. According to the psychoanalytic theory, for instance, happiness is achieved through a sexual pleasure and individuals could escape from the anxiety situations by adopting certain defense mechanisms (Freud, 1910). Humanistic theorists, on the other hand, believe that happiness belongs to those who can accept themselves unconditionally without being judged (Rogers, 1961).

Being ultimate goal of life, when linked to the cultural context, perhaps marriage was one of the universal events across cultures assumed to bring happiness. Growing body of the evidence supports that marriage contributes to individuals’ level of happiness in various cultures (Hirschl, Altobelli, & Rank, 2003; Musick & Bumpass, 2012; Stack & Eshleman, 1998), although many other predominantly Western studies also have been conducted in the last decades to present the opposite finding by highlighting the positive relationship between singleness and happiness (DePaulo, 2013; DePaulo & Morris, 2005). Particularly among most Asians, marriage is also considered important not only for the married couples, but also for their bigger family members (To, 2015; Utomo, Reimondos, Utomo, McDonald, & Hull, 2016). This phenomenon explains why parents often give considerable tensions toward their unmarried children as they reach the marriageable ages (To, 2015; Wang & Abbott, 2013).

Nevertheless, the degree to which marriage increases individuals’ level of happiness is questioned nowadays as more current data follows a path that signifies individuals’ preference to remain single. In the global context, it is evident that more people choose to remain single and to marry older (Australian Bureau of Statistics, 2016; Corselli-Nordblad & Gereoffy, 2015; US Census Bureau, 2016). Within Asia, Jones and Yeung (2014) also noted that marriage has undergone shifting values as more people are favoring single lifestyle.

This paper was particularly focused on the singleness in Indonesia. There are two typical characteristics that lead us to consider Indonesia as the geographic scope of this study. Firstly, the proportion of single people in Indonesia has sharply increased over the years (Badan Pusat Statistik, 2010a; Jones, 2010), whereas the societal acceptance toward singles remains low (Situmorang, 2007), causing singles to be very prone to becoming the target of despising and derogation. This condition is the opposite to what currently occurs in many other Asian countries, which Western values have been well-permeated, such as Singapore. In Singapore, increasing single proportion is accompanied by bigger societal acceptance toward them (Jones, Yanxia, & Zhi, 2012). Moreover, although the single proportion is growing in the last decades, studies regarding singles in Indonesia are still very limited.

Secondly, Indonesia is an ideal setting to examine the relationship between singleness and religiosity. While singles are undervalued in the society, there are indications that people in Indonesia tend to regard positively those who demonstrate religious attributes and symbols (Imanda, 2011; McBeth, 2016). This fact is essential particularly in relation to understanding singles’ coping way in overcoming negative social judgment. Given the fact that religious attributes are positively regarded, singles may be tempted to adopt a certain pattern of religious coping style that may be less beneficial to them as they only use religious demonstrations as a shield to protect themselves from being derided without making spiritual meaning of their singlehood periods.

## Objective

The objective of this examination of the literature was to present the various roles of religiosity in assisting Indonesian singles to overcome both psychological and social burdens due to their single status, relying on the compilation of current findings of related phenomena. For that purpose, there is a need to define the scope of singles. In this paper, singles refer to those who are heterosexual and are never married regardless their motives of being single. The discussion about singlehood in this paper would then be based on this scope.

Despite this paper being focused in Indonesia, the discussion has relevance for other Asian countries which share cultural similarities, particularly considering that singleness is a rising phenomenon in Asia (Jones & Yeung, 2014) and that there is only a few studies conducted regarding singleness within the Asian context. Further, this paper also serves as the first paper from Asian perspective that explores the roles of religiosity in assisting singles and how it implies to the counseling context.

## Method

**Figure 1:**
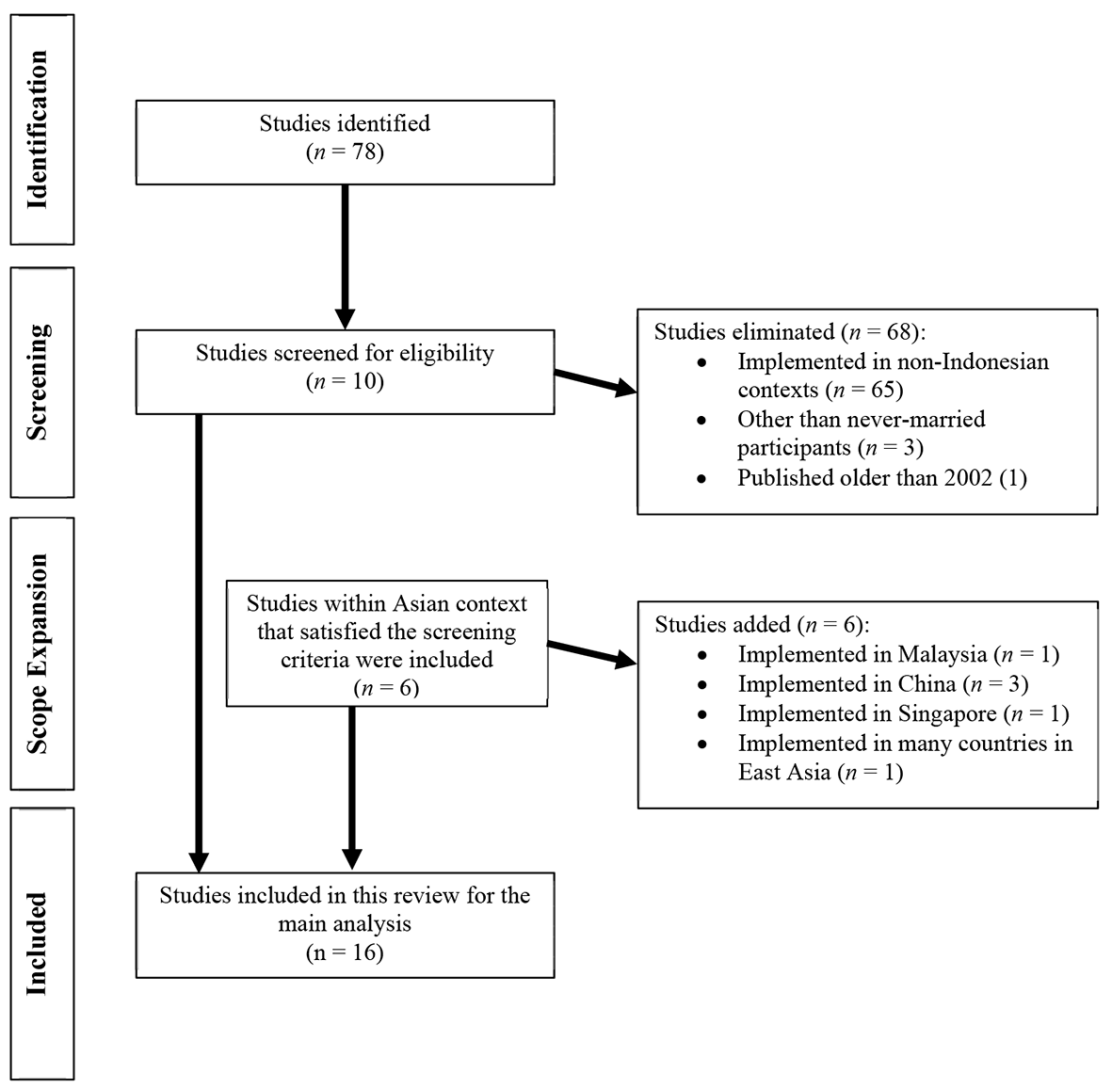
The Process of Studies Selection for the Main Analysis.

This paper presents a systematic compilation of current research findings by conducting a database search strategy. Major databases were included in finding relevant articles, including Scopus, ScienceDirect, PsycArticles, and PsycINFO. Both qualitative and quantitative research articles were included in this review. However, studies that focused on homosexual singles, single parents, or divorced participants were excluded to eliminate biases in the interpretation since this paper focuses primarily on the heterosexual singles. To ensure the currency of the findings, our search was primarily focused on the publication dates of the last 15 years. Given a very few studies ever conducted in Indonesia regarding singlehood, we expanded our search to also include studies within Asian contexts, assuming they have a degree of cultural similarities. Figure 1 presents the process of selection of the studies. Other than those studies, Western-based studies were also included to assist in developing many related concepts (including: modernization and formulation of the single lifestyle) and to enrich the discussion of implications in the counseling setting. 

To provide the context, this paper began by presenting the overview of growing single numbers in Indonesia, the factors that promote the single lifestyle, and the exploration of singles’ perspective toward marriage. The discussion was then focused on the roles of religiosity as a coping way for singles. At the end of this paper, the implications for counselors, social workers, and other relevant mental health practitioners was discussed based on the current findings related to the existing singlehood phenomenon in Indonesia.

## Results

### Singles in Indonesia: The Increased Proportion and Quality of Life

Despite being a country which society regards the universality of marriage (Hull, 2002), the trend toward singleness in Indonesia appears to be consistently increasing over the years. This fact is partially reflected through individuals’ delayed age of first marriage. While in 1970, people started to marry at the age of 19 (Jones, 2010), the most recent census showed that the average age of marriage for males were 25.7 and 22.3 for females (Badan Pusat Statistik, 2010b). The proportion of never-married women aged 35-39 were also increased nearly three times, from only 1.4 percent in 1970 (Jones, 2010) to 3.8 percent in 2010 (Badan Pusat Statistik, 2010b). As many as 6.83 percent of individuals aged 30-39 years were single in 2000 (United Nations, 2017), while in 2010 the proportion increased to 7.18 percent (Badan Pusat Statistik, 2010b).

Notwithstanding the proportion being significantly lower than those in other Asian countries, such as Japan, Hong Kong, Taiwan, or Singapore (Jones & Yeung, 2014), studying singles in Indonesia remains to be significant due to the following reasons. First, being as one of the most populated countries in Asia, when Indonesian singles’ percentage is converted to numbers, there are nearly three million singles (Badan Pusat Statistik, 2010b) whose well-being is likely to be threatened due to being derided by their surroundings. Secondly, with only about seven percent of single adults in the society, it is obvious that marriage is still regarded as a culturally normative event. This situation then creates certain tension for singles to marry in order to comply with the social expectation of them (Jones, 2001; Jones & Yeung, 2014; Situmorang, 2007).

Nevertheless, a recent national survey conducted in 2014 provides a striking evidence regarding singles’ quality of life. In contrast with the previous findings that demonstrate the linkage between marriage and happiness, that survey revealed that when compared to marrieds, never married individuals were found to be significantly happier (Badan Pusat Statistik, 2015). Despite the slight happiness level difference between those groups, this data is imperative to highlight the likelihood of shifting marriage perception among Indonesian society. Although no further explanations were provided in that study regarding in which aspects of happiness singles are outstripping marrieds, many factors, particularly related to the modernization, may explain why more Indonesian adults are choosing to remain single nowadays.

### Modernization and the Single Lifestyle in Indonesia

The increasing rates of singleness cannot be understood without a reference to the variety of social transformations created by modernization. Modernization stimulates series of both social processes, including urbanization, gender equality, higher education, higher job opportunity (Rössel, 2012), and personal values, such as: increased personal efficacy and equality of gender (Hamamura, 2012). In relation to the single lifestyle, the effects of modernization can be grouped into at least four themes: gender equity, shifting personal values, increasing acceptance of marriage-like forms, and impacts of technology (Himawan, Bambling, & Edirippulige, 2017a).

The effects of many changes brought by modernization to individuals’ preference on remaining single can be sociologically explained. Gender equity acts result in the increased women participation in both education and industry (Badan Pusat Statistik, 2016a, 2016b). With more women competing in industries, marriage rates in Indonesia are subsequently reduced because society persists to define an ideal marriage to occur between higher status men and lower status women (this definition of marriage is also known as the hypergamy norm of marriage (Yeung & Hu, 2016)).

Modernization also facilitates many Western values to be permeated in the society, including the values of marriage and the attitude toward sex and dating (Hull, 2002). The demand of industrial development to increase human participation in the workforce stimulates urbanization (Rössel, 2012). More people from rural areas move to city or factory areas and set up a new living arrangement, which also includes a formation of a new set of values that emphasizes individualism and self-reliance (Sachs, 2005). They are also more likely to be exposed with various marriage-like arrangements, such as cohabitation, that offer emotional and sexual enjoyment without necessarily being committed in the long run (Raymo, Park, Xie, & Yeung, 2015). Despite the practice of cohabitation is not acknowledged both by the Indonesian law and the society (Fachrudin, 2016), it begins to be a common practice in the recent years with an increased acceptance by the younger generation (Rahyani, Utarini, Wilopo, & Hakimi, 2012). In addition to that, online porn materials may be seen as an acceptable way of sexual gratification among singles. The sexual content over the internet has been found to alter adults’ sexual needs through various methods from text message to video sex (Drouin, Vogel, Surbey, & Stills, 2013), providing a channel for sexual stimulation without necessarily have to be in the long-term relationship. Further, such use of technology to access porn materials is also indicated to be a factor that reduces marriage likelihood (Malcolm & Naufal, 2014), which occurs as a result of technological modernization.

### Indonesian Singles’ Attitude Toward Marriage

Despite the fact that singles are growing both in numbers and in their quality of life and that many factors contribute to the increasing favorability of single lifestyle, it appears that apparently most Indonesian singles are still desiring marriage. In this part, some evidence related to Indonesian singles’ attitude toward marriage is presented.

A qualitative study conducted by Situmorang (2007) suggests that the predominant reason for individuals to remain single is because they have not yet met their potential partners. This finding is also articulated by Vignato (2012) that highlights the interaction between sexual passion and religious compliance in determining singles’ marriage preference. In both studies, singles were described as being involuntarily single.

A study has been done to explore the degree of marriage favorability among Indonesian singles (Himawan, 2017). Participated by 107 singles, it was found that more than 80 percent of them indicated their intention to marry. Further, although more than 90 percent of them reported being distressed due to excessive marriage pressures received from their surroundings, most of them expressed their reasons to marry beyond for the sake of social compliance. Their three predominant reasons to marry are as follows: (1) for the emotional fulfillment (38.95%): to make life complete, to gain support and affection, to share life burdens, to gain personal enjoyment; (2) for procreation purposes (28.42%): to have an offspring, to build a family; and (3) for spiritual fulfillment (13.68%): to fulfill God’s calling, to obey to God’s demand.

All of the above studies show a strong indication that most of the Indonesian singles are involuntarily singles. A particular attention regarding singles’ effective coping strategies is therefore instructive to be drawn considering that their well-being is likely to be threatened due to their involuntary status.

### How Singles Compensate Their Need to Attach

A predominant feeling singles, particularly involuntarily singles, may experience is a feeling of loneliness (Wang & Abbott, 2013). This could be understood because every human has a fundamental need to belong (Baumeister & Leary, 1995). Baumeister and Leary (1995) argue that a relationship that can satisfy ones’ need to belong is characterized by three components: (1) provides a meaningful interpersonal connection, (2) enables frequent physical interaction, and (3) fosters the sense of security. Among adults, this relationship is perhaps best manifested through marital relationship. However, in a case where a romantic partner is absent, as in the case of singles, many potential alternative relationships are available, including: relationship with parents, family members, friends (Morris & DePaulo, 2009), or through a spiritual connection with God and religious communities (Kirkpatrick, 1992).

Many Western-based studies point out the effective roles of social support from family and friends among singles (DePaulo & Morris, 2005; Morris & DePaulo, 2009), however limited studies are available to sufficiently conclude such effectiveness in both Asian and Indonesian context. In light of the limited empirical evidence, a comparative study of single women in Indonesia, Thailand, and Philippine showed that Indonesian single women seem to take more passive roles in the family and are reluctant to share their personal problems with family members (Tan, 2010). Some singles are also preferred to be self-reliant in solving their personal problems by choosing to build a meaningful spiritual relationship rather than sharing with their friends or family members. This finding articulates the assumption of important roles of religiosity in assisting singles to overcome their challenges.

### The Roles of Religiosity in Overcoming Singles’ Psychological and Social Challenges

Religiosity may offer a promising outcome for singles since it facilitates both psychological attachment and accepting community (Saroglou, 2011) to which singles may belong. Being lonely and burdened due to negatively valued, singles may be benefited through building spiritual attachment with a Higher Being. The idea of religion is originated based on human feelings of fear and loneliness, and therefore spiritual attachment operates on the function to satisfy the human need for security and belongingness (Kirkpatrick, 1992). Through building an intimate interaction with the Higher Being, individuals begin to gain a meaning behind their current situation (Saroglou, 2011). That spiritual meaning would make individuals feel more secure as they understand that their current condition does not stand for itself but rather is served for bigger and transcendent purposes (Pargament et al., 1990). Whereas the attachment need can be provided through romantic relationships (Schachner, Shaver, & Gillath, 2008), in the case of singles, Kirkpatrick (1992) believes that religiosity could serve a similar function when partners are not available.

Himawan, Bambling, and Edirippulige (2017b) concluded that singles could be benefited from adopting adaptive religious coping way, which is indicated by: (1) the focus on the values rather than symbols of their religions (as according to Allport’s (1966) concept of religious orientation), (2) the less tendency to perceive God as the punishing figure (as according to Pargament, Feuille, and Burdzy (2011)), and (3) the ability to build and maintain relationship with the Higher Being and with their religious community (as according to Saroglou, 2011). By practicing this kind of religious coping, not only that singles could draw meaning of their singlehood as something that helps develop their spirituality, they also could be benefited through their involvement in the religious community, particularly if their religion does not strongly oppose the position of being singles. Motives of being singles also determine the degree to which they are accepted within their religious communities because religious singles tend to define their singlehood as temporary period and not as a personal choice (Engelberg, 2016; Ibrahim & Hassan, 2009). Such motives could increase more sympathetic reaction from their communities (Slonim, Gur-Yaish, & Katz, 2015). Generally, although they may be negatively judged by general society, the religious community offers more trusting and accepting atmospheres (Dingemans & Ingen, 2015), in which they could engage without being undervalued.

However, adopting the religious approach in assisting singles should be performed cautiously, particularly in the Indonesian context. Pargament and Park (1995) concluded that maladaptive religious coping is manifested in the form of denial, passivity, and resistance to social change. Denial of the reality occurs when singles are preoccupied practicing extrinsic religiousness (as described by Allport, 1966), in which they deny their real burdens associated with being singles by articulating themselves with religious attributes and rituals. Further, when singles are adopting the pessimist view of religion, such as by seeing the Higher Being as punishing figure (Pargament et al., 2011), they are more likely to be passive in their current situation and to be resistant to change. In addition to that, when focused on the substantial content of most religious beliefs, singles may also feel burdened of practicing religiosity, especially when they emphasize how their religious teachings encourage them to marry. Ample of research have demonstrated that singles whose religion encourage them to marry are struggled due to their inability to comply with their religious demands (Darrington, Piercy, & Niehuis, 2005; Engelberg, 2016; Ibrahim & Hassan, 2009). This suggests that religiosity may also cause singles to feel more inadequate, which leads to their lower level of well-being.

Further, when putting religiosity into the perspective of Indonesian culture, maladaptive religious coping may be more likely to occur considering that the society often tends to regard those with the religious demonstration in an overly positive manner (Imanda, 2011). Religiosity is considered to be a very fundamental aspect among Indonesian society to the extent that religious and culture based thinking are pervasive among the people, regardless their education and ethnicities (Himawan, 2014). Being regarded very positively, attaching themselves with many religious attributes without taking an advantage of making spiritual meaning in reflecting their involuntary status could be a shortcut way for singles to be less stigmatized (Himawan et al., 2017b). In this case, singles would likely be burdened practicing such maladaptive religious coping way. Practicing such maladaptive coping could lead to an identity dissonance due to the fact that they reflect themselves to be highly religious while also being single (Engelberg, 2016). Many studies demonstrate how those who are preoccupied heavily on religious rituals and symbols are likely to experience negative psychological outcomes (Doane, Elliott, & Dyrenforth, 2014; Navara & James, 2005; Power & McKinney, 2014; Tahmasbipour & Taheri, 2011).

## Discussion and Implications for Counselors

There are social and psychological risks associated with being singles in Indonesia. The challenges are intensified by the fact that, whereas most Indonesian singles are involuntarily singles, the society often attributes singleness with social failure and incompetence. Such interplay between societal pressure and something that goes beyond individual control creates a condition that definitely threatens singles’ well-being.

Examining the roles of religiosity in assisting singles is relatively a new area of study and therefore very limited resources are available to provide the empirical based relationship between religiosity and singles’ well-being. After reviewing the available literature, however, this paper has highlighted the key finding regarding the roles of religiosity as singles’ coping way. It could be concluded that singles’ motives of practicing religiosity would determine whether the outcome would be promising or detrimental. Despite the phenomenon being understudied, this paper provides a basis and a direction for future relevant studies regarding the use of religiosity as one of the coping ways for singles. More empirical studies are certainly needed to provide evidence based arguments regarding the role of religiosity in assisting singles, especially in Asian context, in which cultural and religious values are still well-permeated in the daily practice. On the practical ground, the roles of government and other related parties are essential to modify societal perception towards singles, particularly with a notion that most Indonesian singles are actually having an intention to marry.

Counselors (and psychologists; due to counseling sessions in Indonesia are also commonly performed by psychologists) hold a vital role in assisting singles to overcome their psychological and social challenges. With a low yet constantly rising psychological awareness in Indonesia (Setiyawati, Blashki, Wraith, Colucci, & Minas, 2014), it is reasonable to expect that counseling sessions around singlehood theme will continue to arise as single proportion is consistently increasing and there is no observable government and social strides to reduce stigma toward them. Therefore, counselors need to have a good understanding of psychological and social dynamics of singles, particularly in relation to the social and cultural contexts.

In assisting singles, counselors need to be aware of their own attitude toward marriage that may intervene the counseling sessions. Counselors are urged to provide a non-judgmental atmosphere in the counseling sessions, which is a required condition to enable singles to securely disclose their personal problems. Therefore, whether counselors are pro-marriage or singlehood, they must address and position clients’ attitude toward marriage as the utmost goal of the sessions. An understanding of singles’ motives (whether they are voluntarily or involuntarily singles) could be one of the ways to assess their readiness toward perceived societal judgment, which would add more insight in formulating appropriate counseling strategies.

With regards to the religiosity, despite the fact that the religious coping may be beneficial for singles, counselors need to have good knowledge and skill in integrating religious issues in the counseling sessions. Many counselors tend to maintain a secular point of view in their sessions and thus attempt to eliminate clients’ religious values (Lothstein, 2002). Nevertheless, in the last two decades, the trend toward integrating spiritual and religious issues in the counseling session emerges and more clients prefer to receive services from counselors who are sensitive to religious issues (Sperry, 2012). When addressing religious issues in the counseling sessions, it is worth noticing counselors’ approach to religiosity as categorized by Zinnbauer and Pargament (2000) (Rejectionist, Exclusivist, Constructivist, or Pluralist), so that appropriate actions could be taken accordingly (such as: referring clients, adjusting clients expectation in the counseling sessions) should there is a mismatch between counselors’ approach to religiosity and clients’ condition.

Lastly, although this paper demonstrates the possible effective roles of religiosity in assisting singles, it does not necessarily mean that religious approach must be applied when handling never married clients, especially when handling atheist single clients or clients who prefer to argue on the secular or rational basis. The roles of religiosity are most effective for clients who identify themselves in certain religious beliefs. Counselors should encourage clients in practicing religiosity according to their beliefs, rather than insisting counselors’ religious beliefs. Given this situation, client referrals perhaps could be a good solution when friction occurs as a result of the discrepancy between the religious values of both counselors and the clients.
